# Role of FKBP5 and its genetic mutations in stress-induced psychiatric disorders: an opportunity for drug discovery

**DOI:** 10.3389/fpsyt.2023.1182345

**Published:** 2023-06-16

**Authors:** Mahdi Malekpour, Dorsa Shekouh, Mohammad Ebrahim Safavinia, Shadi Shiralipour, Maryam Jalouli, Sahar Mortezanejad, Negar Azarpira, Niloofar Dehdari Ebrahimi

**Affiliations:** ^1^Student Research Committee, Shiraz University of Medical Sciences, Shiraz, Iran; ^2^Transplant Research Center, Shiraz University of Medical Sciences, Shiraz, Iran

**Keywords:** FKBP5, psychiatric diseases, stress induced psychiatric disorders, FKBP5 genetic, drug discovery

## Abstract

Stress-induced mental health disorders are affecting many people around the world. However, effective drug therapy for curing psychiatric diseases does not occur sufficiently. Many neurotransmitters, hormones, and mechanisms are essential in regulating the body's stress response. One of the most critical components of the stress response system is the hypothalamus-pituitary-adrenal (HPA) axis. The FKBP prolyl isomerase 51 (FKBP51) protein is one of the main negative regulators of the HPA axis. FKBP51 negatively regulates the cortisol effects (the end product of the HPA axis) by inhibiting the interaction between glucocorticoid receptors (GRs) and cortisol, causing reduced transcription of downstream cortisol molecules. By regulating cortisol effects, the FKBP51 protein can indirectly regulate the sensitivity of the HPA axis to stressors. Previous studies have indicated the influence of FKBP5 gene mutations and epigenetic changes in different psychiatric diseases and drug responses and recommended the FKBP51 protein as a drug target and a biomarker for psychological disorders. In this review, we attempted to discuss the effects of the FKBP5 gene, its mutations on different psychiatric diseases, and drugs affecting the FKBP5 gene.

## 1. Introduction

A mental health disorder may be defined as a condition in which a person experiences abnormal thoughts, perceptions, feelings, behaviors, and distress (1). An estimated one billion people worldwide suffer from mental illness, and projections indicate that this number will continue to rise (2). Every 40 s, a suicide occurs, and ~160 million individuals require humanitarian assistance due to mental health concerns (3). Individuals suffering from mental health issues often experience a compromised quality of life marked by diminished self-control, impaired judgment, reduced self-assurance, feelings of demoralization, and hopelessness (4).

Despite many people being affected by psychiatric disorders, there is a substantial lack of effective cures for psychiatric diseases (5–8). The etiology of psychological manifestations is often unclear, particularly concerning molecular aspects (5, 6, 9). However, despite the availability of monoaminergic anti-depression drugs, less than half of the patients experience full rehabilitation after taking medications (5). Furthermore, pharmacogenetic studies have highlighted the associations between an individual's genetic variations and treatment outcomes. Therefore, pharmacogenetic findings indicate the potential for more effective molecular interventions (10). These pharmacogenetic studies, alongside twin studies, have highlighted the importance of genetic and molecular factors in psychiatric diseases (10, 11). Moreover, approximately one-third of schizophrenic patients are diagnosed as “treatment resistant” (12). Psychiatric treatment resistance is characterized by a correct diagnosis, sufficient and suitable treatment, and an inadequate symptomatic response. Treatment resistance is currently defined in a variety of psychiatric diseases, including obsessive-compulsive disorder (OCD) (13), bipolar affective disorder (14), schizophrenia (15), and major depressive disorder (MDD) (16, 17). The number of published studies indicates the growing rate of treatment resistance year by year (18). Under some circumstances, a patient's specific condition can create barriers to achieving the full potential of treatments. For instance, in both MDD and schizophrenia, liver drug metabolism (19, 20), tobacco smoking (21, 22), and genetic variations are predisposing factors to inadequate response to treatment (18). Furthermore, pharmacogenetic studies have demonstrated the relationships between an individual's genetic variations and treatment outcomes. Thus, as a consequence, pharmacogenetic findings could be applied to more effective molecular interventions (10). One of the mechanisms that are considered to potentially trigger psychological disorders underlying psychoneuroendocrinology is hormone and transmitter fluctuation in the central nervous system (CNS) (23). Endocrine-produced steroid hormones can passively enter the CNS, acting as a transcriptional factor leading to the upregulation of some genes, neuron excitability modification, and mood and behavior alterations (24). The glucocorticoid receptors (GRs) are omnipresent and expressed in almost all cells, including CNS cells. Previous studies have shown that GR expression in specific CNS parts could regulate mood and behavior (25). Dysregulation of steroid hormone receptors, such as the upregulation of the hypothalamus-pituitary-adrenal (HPA) axis, can lead to mood disorders such as depression (26). In this regard, restoring factors such as FK-506 binding proteins 51 (FKBP51) and 52(FKBP52) can be promising targets (27).

FKBP51 acts as an inhibitor for GRs' translocation to the cellular nucleus and is also associated with the negative feedback mechanism of these receptors. GR activation affects both inhibitory and excitatory synapses in the hippocampus. However, this regulatory function is dependent on the presence of FKBP51, as evidenced by studies indicating that such mediation does not occur in its absence (28). Additionally, Genomic Wide Association Studies (GWAS) have revealed a correlation between certain allelic variants of FKBP5 and various mental health conditions, including but not limited to aggression (29), bipolar disorders (30), suicide (31–34), post-traumatic stress disorder (PTSD) (35), negative personality traits (36), and peritraumatic dissociation (37). An animal study on mice indicated that FKBP5 may have a crucial role in morphine addiction induction (38). FKBP5 also has a significant association with a higher probability of severe depressive disorder in subjects with methamphetamine use disorders (39). Besides some other genes, FKBP5 has an effect on the electroconvulsive therapy effect in resistant major depression cases (40). Recently, an association between mutations of FKBP5 and functional seizures, another stress-induced disease whose biological aspects are not well known, has been found (41, 42). Moreover, in another study, potential drugs for targeting the FKBP51 protein have been investigated and reported (43) because the inhibition of FKBP51 can promote the regulation of the HPA axis in stress-induced disorders (41, 43). In this study, we attempted to explain the role of FKBP5 in the human body, especially in the HPA axis and stress-induced disorders, the effects of the most important genetic mutations of this gene in stress-induced disorders, and the potential therapeutic effects affecting FKBP51.

## 2. FKBP5's mechanism of action

The FKBP5 gene is located on chromosome 6 (6p21.3) in humans and consists of 13 exons. The FKBP5 gene encodes the FKBP51 protein, a member of the immunophilin protein family (44), which derives its name from its capacity to bind to immunosuppressive drugs (45). The peptidyl-prolyl isomerase (PPIase) domain, present in all members of this family, allows them to bind to different drugs such as rapamycin, cyclosporine A (a subfamily of cyclophilins), and FK506 (an FKBP subfamily protein), based on their ability to recognize them selectively (45, 46).

The FKBP51 and FKBP52 proteins of the immunophilin family are named for their high molecular weights (51 and 52 kDa, respectively) (47). FKBP51 and FKBP52 are homologous and share 75% similarity. The remaining 25% difference between these two proteins potentially affects their conformation and domain orientation and, consequently, their divergent effects on steroid hormone receptor (SHR) activities (48, 49). Further, FKBP51 and FKBP52 contain tetratricopeptide-repeated (TPR) domains, which allow them to bind to heat shock protein 90 (HSP90) dimers (50). The HSP90 protein plays an important role in the maturation and folding of SHRs, allowing them to bind to their ligands (51). Furthermore, the FKBP51 protein functions as a negative regulator of GRs in association with the HPA axis (52). As a substantial regulatory factor of the HPA axis, GR (Nuclear Receptor Subfamily 3 Group C Member 1 (NR3C1) gene) is a ligand-dependent transcription factor that modulates gene transcription (activation or repression) in the nucleus for diverse genes, including the FKBP5 gene (53–55). GCs, by interacting with GRs, can induce stress-related actions such as vasoconstriction, lipolysis, and suppression of reproduction, preparing the human body for “fight or flight” actions. Chronic stress can disturb the resilience of neurons, causing susceptibility to many human psychiatric disorders (56). This is the reason for the importance of the negative regulatory effects of FKBP51 on GRs.

**Figure 1:**
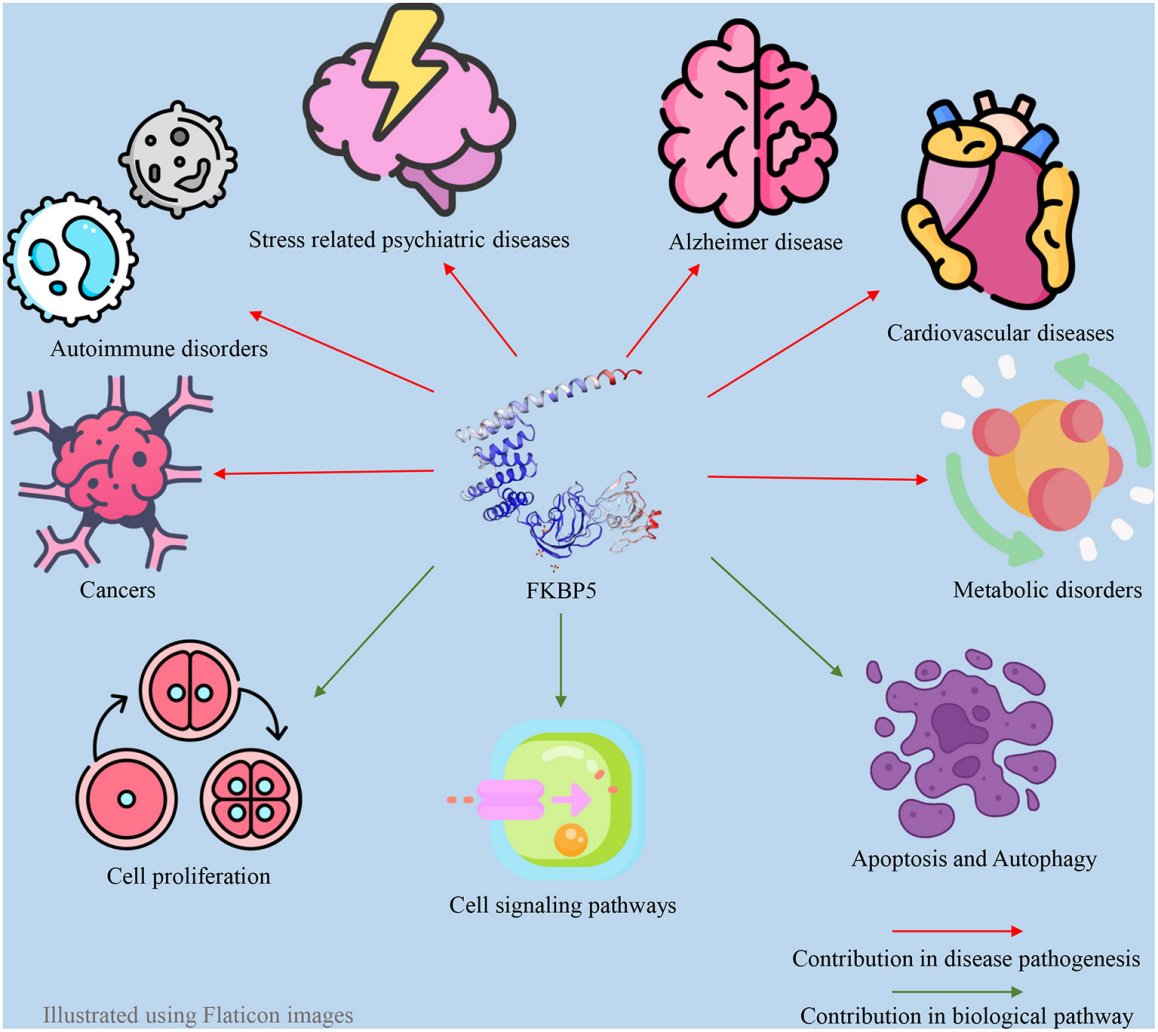
Contribution of FKBP5 in different pathophysiologic aspects of the human body.

FKBP51 plays a pivotal role as a regulatory factor in numerous biological processes within both the central and peripheral nervous systems. FKBP51 exerts its effect through a variety of direct and indirect mechanisms and Hsp90-dependent or Hsp90-independent pathways (57). The impact of FKBP51 on critical cellular signaling pathways is well established, such as the modulation of Akt and the mammalian target of rapamycin (AKT/mTOR) pathway (58), Tau formation (59), nuclear factor kappa B (NF-κB) pathway (60), microtubule dynamics (61), epigenetic remodeling (62), metabolism (63), apoptosis (64), autophagy (64), and reproduction (27). Thus, changes in FKBP51 expression and function are associated with the occurrence of a wide variety of disorders, including metabolic syndrome (65), Alzheimer's disease (66), the development of different types of cancer, cardiovascular disease, and immune disturbances, in addition to stress-induced psychiatric disorders (67, 68). FKBP5 also has an association with atrial fibrillation. Indeed, the loss of function of the FKBP5 gene has a significant impact on atrial fibrillation development and cardiac function (69). In addition to responding to stress, the HPA axis can regulate sleep mechanisms via the FKBP5 gene. As a result, a disturbed FKBP51 protein level can act as a pathogenic factor for sleep disturbance (70). Additionally, since androgens and androgen receptors (ARs) directly and indirectly regulate the FKBP5 gene, this gene may also be associated with polycystic ovary syndrome (PCOS) and hyperandrogenism (71). These associations between the FKBP5 gene and various diseases make FKBP51 a promising drug target. For example, FKBP5 is a target for anti-depressants and anti-cancer drugs. Furthermore, FKBP51 may serve as a biomarker for cancer diagnosis and chemotherapy response in patients with varying levels of FKBP51 changes (67, 72, 73). Many studies have indicated a significant negative association between FKBP5 and the severity of different types of cancer (73–76). Furthermore, previous studies have indicated that FKBP51 could be a potential diagnostic marker for psychiatric diseases (77, 78). Some of the roles of FKBP51 in different aspects of human physiology can be observed in Figure 1.

The HPA axis is a neuroendocrine stress response system activated by exposure to physical or psychological stress. The HPA axis regulates many human body systems, including the metabolic, cardiovascular, and immune systems, resulting in adaptation to the human environment (56). In more detail, stress triggers the hypothalamus to release the corticotropin-releasing hormone (CRH), which stimulates the pituitary gland to produce adrenocorticotropic hormone (ACTH). ACTH stimulates the adrenal glands to release glucocorticoid hormones (GCs), such as cortisol, which circulate throughout the body and bind to GRs, leading to negative feedback control (65, 79). Besides, in the cells, GR and other regulatory proteins, including HSP90, make a protein complex which bindes to cortisol. The role of regulatory proteins is to change the formation of GR to a conformation with more affinity to bind hormones (27). The activated GRs with cortisol induce the transcription and translation of the FKBP5 gene (57). FKBP51 expression is regulated by the interaction of GR with glucocorticoid response elements (GREs) (44). These enhancer elements can bind to the FKBP5 gene in the 2, 5, and 7 introns and the promoter, which are regions enriched with cytosine phosphate guanine (CpG) islands (80). The FKBP51 protein can inhibit GR function through a negative feedback loop (44, 81). This inhibition occurs through a complex interplay involving the replacement of FKBP51 with FKBP52 in the receptor heterocomplex and the modification of GR folding and conformation. As a result, the affinity of GR for cortisol decreases, leading to GR resistance and an increase in circulating cortisol levels.

**Figure 2:**
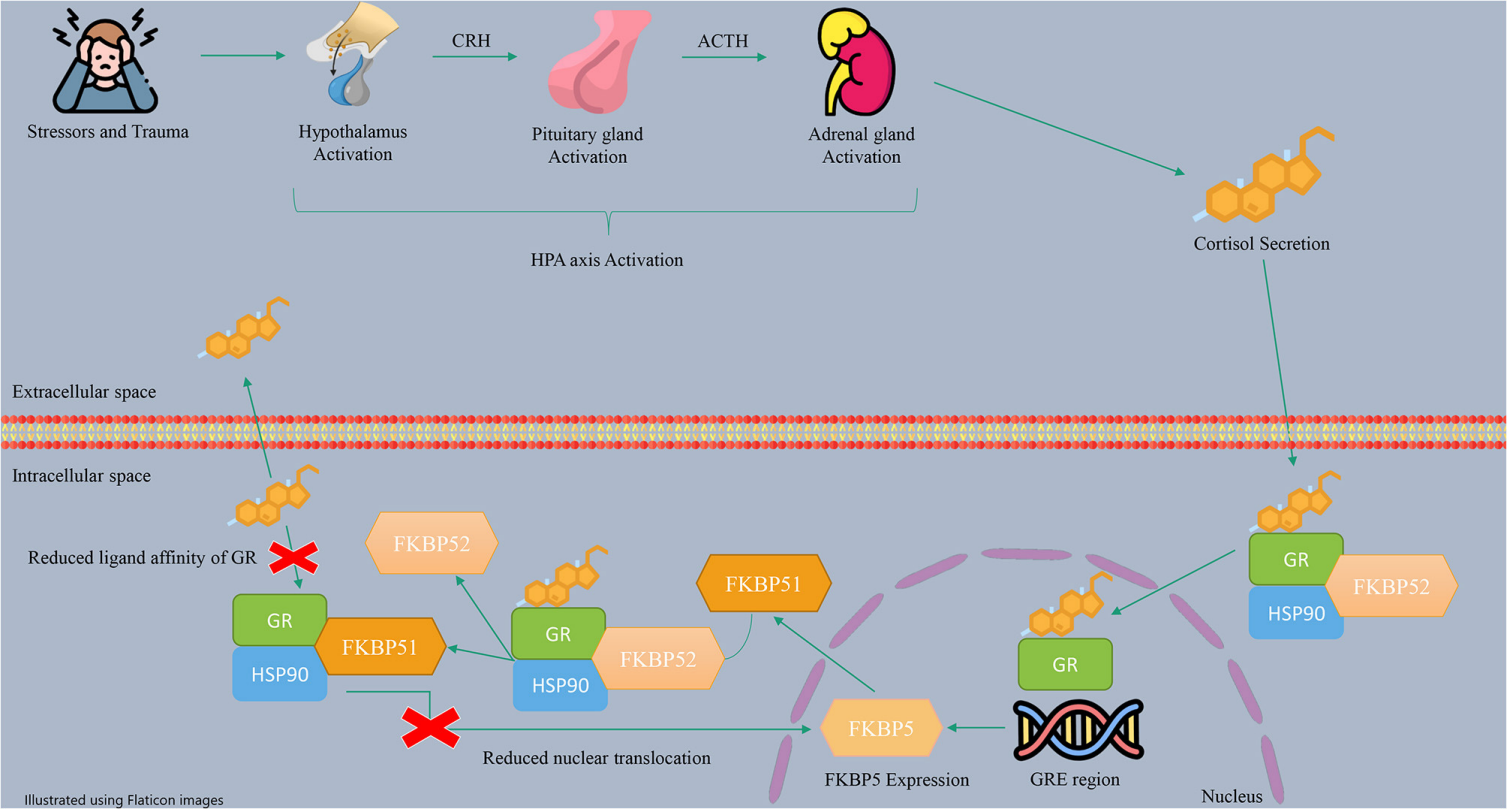
The molecular pathway of FKBP5 effects on the HPA axis.

Additionally, by enhancing the FKBP51 level, FKBP51 can inhibit the translocation of GRs to the nucleus, resulting in a reduction in the expression of GR target genes (82, 83). Therefore, FKBP51 helps maintain HPA axis balance during periods of stress. A summary of the described mechanism of action of the HPA axis and FKBP5 is shown in Figure 2.

The HPA axis and its regulators are intricately connected; any imbalance among these components may result in psychiatric disorders (84). Any dysregulation of the HPA axis caused by genetic, epigenetic, or environmental factors may result in the altered expression of the FKBP51 protein (44, 85, 86). However, genetic and epigenetic variations of the GREs and the FKBP5 gene can influence the expression level of the FKBP51 protein (80). Some single nucleotide polymorphisms (SNPs) of GREs and the FKBP5 gene are associated with increased expression of FKBP51, while others are associated with a decreased expression of FKBP51 (87, 88). There is a positive correlation between these changes in FKBP5 gene expression levels and mental disorders (28).

In terms of translation regulation, it has been demonstrated that DNA methylation is the most common epigenetic modification studied for FKBP5 compared with post-translational modifications and non-coding RNAs. In CpGs, the methylation process contributes to chromatin compression and usually reduces gene expression by inhibiting transcription factor binding. It has been demonstrated that long-lasting methylation of genes associated with the glucocorticoid signaling pathway may alter the function of these genes and contribute to a variety of mental health disorders, particularly during early-life prolonged stress conditions and childhood adversity (89, 90). Additionally, methylation of FKBP5 leads to accelerated DNA methylation aging and, consequently, cardiometabolic risk (91). Recent studies have shown that FKBP51 plays a direct role in epigenetic processes by controlling phosphorylation and activating the DNA methyltransferase 1 (DNMT1) enzyme (62). Genetic polymorphisms and DNA methylation of FKBP51 proteins are transcriptional modifications (92, 93). The expression of FKBP51 can also be regulated at the post-transcriptional level through interference with FKBP51 mRNA by micro-RNAs (94) and post-translational modifications such as protein phosphorylation (95). Studies have shown that micro RNAs (miRNAs) and the FKBP5 gene interact at a posttranscriptional level. For example, non-coding RNA molecules such as circular RNAs (circRNAs) and long non-coding RNAs (lncRNAs) can contribute to the progression of autoimmune diseases and cancer through miRNA/FKBP5 axis regulation (96, 97).

## 3. FKBP5 genetic variations and psychiatric diseases

The HPA axis is one of the most important biological components of the stress response (98, 99). Thus, genetic factors regulating the HPA axis may affect individual HPA axis responses and stress-related psychological issues (100, 101). Many studies have examined the effects of genetic polymorphisms and epigenetic modifications on the HPA axis' response to stress (102). In this regard, FKBP5 has attracted much interest due to its regulatory effect on the HPA axis by regulating GC sensitivity (103). Variants of the FKBP5 gene can cause dysregulation of the stress response in healthy individuals through decreased negative feedback efficiency between FKBP5, GRs, and GCs, as well as the dysregulation of GR sensitivity (87, 88). In this section, we have attempted to demonstrate the association between the most studied SNPs of the FKBP5 gene and various mental problems. Information concerning SNP regions in the human genome and the frequency of each allele was obtained from the database of SNPs (dbSNP) and is presented in Table 1 (104). Additionally, a summary of relevant studies investigating the association between FKBP5 SNPs and different mental health conditions has been provided in Table 2.

**Table 1:** The overview of the FKBP5 SNPs.

**SNPs**	**Position**	**Functional consequence (gene: consequence)**	**Frequency of alleles in healthy individuals**
Rs1360780	chr6:35639794	Intron variant (LOC112267956: intron variant)	*T* = 0.31417 *C* = 0.68583
Rs9296158	chr6:35599305	Intron variant (LOC112267956: 2KB upstream)	*A* = 0.312860 *G* = 0.687140
Rs4713916	chr6:35702206	Intron variant	*A* = 0.30384 *G* = 0.69616
Rs7748266	chr6:35624967	Intron variant	*T* = 0.17008 *C* = 0.82992
Rs7757037	chr6:35580459	Intron variant (LOC101929309)	*G* = 0.527180 *A* = 0.472820
Rs9470080	chr6:35678658	Intron variant	*T* = 0.335162 *C* = 0.664838
Rs3800373	chr6:35574699	3 prime UTR variant (LOC101929309)	*C* = 0.288538 *A* = 0.711462
Rs9394309	chr6:35654004	Intron variant	*G* = 0.29378 *A* = 0.70622
Rs4713902	chr6:35646249	Intron variant (LOC112267956)	*T* = 0.741398 *C* = 0.258602
Rs9470079	chr6:35675286	Intron variant	*G* = 0.82382 *A* = 0.17618
Rs3798347	chr6:35633999	Intron variant (LOC112267956)	*A* = 0.32192 *T* = 0.67808
Rs10947563	chr6:35685660	Intron variant	*G* = 0.28036 *A* = 0.71964

**Table 2:** The association between FKBP5 SNPs and different psychiatric diseases.

**Psychiatric disorder**	**FKBP5 SNP**	**Allele**	**Type of association**	***P*-value**	**References**	**Number of participants**
Maladaptive emotional behavior	rs1360780	T	Increased risk of psychiatric disorders	N/A	Halldorsdottir et al. (105)	114 homozygous CATT 530 heterozygous CATT 701 homozygous non-carrier
	rs9470080	T		N/A		
	rs9296158	C		N/A		
	rs3800373	A		N/A		
Neuroticism	rs9470080	T	Increased risk	0.006	Mihaljevic et al. (101)	52 cases vs. 51 controls
Mood disorders	rs4713916	A	Increased treatment response to drugs	0.01	Zou et al. (106)	Meta-analysis of three studies with 1,101 subjects
Bipolar disorder	rs4713902	T	Increased risk	0.0001	Willour et al. (30)	1,188 subjects
	rs7757037	A	Increased risk	0.001		
	rs3800373	G	Increased risk	0.0046	Calabrò et al. (107)	131 subjects vs. 65 controls
Unipolar depression	rs1360780	T	Increased anti-depressant treatment response	0.01	Stamm et al. (108)	127 cases vs. 171 controls
	rs3800373	A, C	Increased risk	0.014	Zobel et al. (134)	268 cases vs. 284 controls
Depression	rs4713916	N/A	Increased risk of depression	N/A	Hernández-Díaz et al. (109)	A meta-analysis of 4 studies with 2,614 participants
	rs3800373	C	Increased risk of depression	N/A		
	rs3800373	C	More frequent in depressed subjects	N/A	Wang et al. (110)	A meta-analysis of 14 studies with 15,109 participants
	rs1360780	T	Increased risk under childhood adversity	< 0.0001		
	rs9470080	N/A	Robust associations	N/A		
	rs3800373	N/A	Robust associations	N/A		
	rs9470080	G	Increased risk of depressive symptoms	0.037	Velders et al. (111)	A meta-analysis of 4 studies with 3,550 participants
	rs1360780	T	Increased recurrences of depressive episodes	0.019	Shibuya et al. (36)	449 male vs. 377 female
MDD	rs4713916	A, G	Increased risk	0.038	Szczepankiewicz et al. (112)	218 cases vs. 742 controls
	rs9470080	T	Increased risk	0.007		
	rs1360780	T	Increased risk	0.011		
	rs9296158	A	Increased risk	0.03		
	rs9394309	A	Increased risk	0.018		
	rs3800373	C	Increased risk	0.002	Rao et al. (113)	12,491 cases vs. 14,091 controls
	rs3800373	C	Increased risk	0.037	Tatro et al. (114)	48 cases vs. 12 controls
	rs1360780	T	Increased risk	0.05	Mikolas et al. (115)	85 cases vs. 67 controls
	rs1360780	T	Lower in MDD patients	0.04	Arlt et al. (116)	44 cases vs. 202 controls
Combined PTSD-MDD	rs9470080	T	Increased risk after low-level trauma exposure	0.003	Li et al. (117)	586 low-level trauma vs. 554 high-level trauma cases
PTSD	rs1360780	T	Decreased risk	0.037	Kang et al. (118)	123 cases vs. 116 controls
	rs9296158	A	Increased risk	0.01	Hawn et al. (119)	A meta-analysis of 9 studies with 8,511 participants
	rs3800373	A	Increased risk	N/A	Zhang et al. (120)	772 women vs. 360 men
	rs9470080	T	Increased risk	0.05		
	rs9470080	C	Increased risk	0.001	Zhang et al. (121)	341 cases vs. 3514 control
	rs3800373	A	Increased risk	0.004		
	rs3800373	C	Interacted with early-life stress to predict higher risks for PTSD	< 0.001	Wang et al. (110)	A meta-analysis of 14 studies with 15,109 participants
	rs1360780	T		< 0.001		
	rs9470080	T		< 0.001		
Chronic PTSD	rs1360780	T	Increased risk	0.037	Kang et al. (118)	123 cases vs. 116 controls
Anxiety disorders	rs1360780	T	Increased risk	< 0.001	Minelli et al. (122)	657 cases vs. 462 controls
	rs9470080	T	Increased risk	0.015	Bryushkova et al. (123)	234 cases vs. 233 controls
	rs4713916	A	Increased risk in women	< 0.001	Isaksson et al. (124)	2,029 males vs. 2,348 females
Impulsive behavior	rs1360780	T	Reduce impulsivity in intertemporal choice	0.047	Kawamura et al. (125)	91 healthy Japanese people
ADHD	rs9470080	C	Increased risk	0.022	Isaksson et al. (126)	81 children with ADHD vs. 88 controls
	rs7748266	C	Increased risk	0.019		
	rs9394309	A	Increased risk	0.013		
Schizophrenia	rs9470080	C	Increased risk	0.044	Mihaljevic et al. (101)	52 cases vs. 51 controls
	rs9296158	A	Increased risk	0.03		
	rs3800373	G	Increased risk	0.041		
Suicide	rs1360780	T	Increased risk of suicide	N/A	Hernández-Díaz et al. (109)	Meta-analysis of 3 studies with 751 cases and 703 controls
	rs4713902	G	Increased risk	0.023	Roy et al. (31)	198 cases vs. 682 controls
	rs3800373	C	High-induction allele	0.007	Fudalej et al. (127)	563 cases vs. 475 controls
	rs1360780	T	Increased frequency of suicide	0.026		
Drug abuse	rs1360780	T	Increased risk	0.04	Levran et al. (128)	852 cases vs. 238 controls
	rs3800373	A	Increased risk	0.03		
	rs3800373	A, C	Increased risk of addiction	0.03	Levran et al. (129)	595 subjects vs. 208 controls
	rs1360780	C, T	Increased risk of addiction	0.04		
Borderline personality disorder	rs9470079	A	Increased risk	0.01	Martín-Blanco et al. (130)	481 subjects vs. 442 controls
	rs4713902	C	Decreased risk	0.03		
	rs3798347	T	More frequent in emotionally neglected cases	0.03		
	rs10947563	A		0.02		
Antisocial personality disorder	rs1360780	T	Increased risk	0.035	Suchting et al. (131)	48 participants before and after giving 20 mg of cortisol

### 3.1. Rs1360780

Anxiety can cause the upregulation of FKBP5, resulting in a lowered affinity of GRs to GCs when sustained, leading to clinical symptoms of anxiety and MDD (122). Observations have shown that emotionally neglected or abused children with the T allele of rs136780 have lower DNA methylation in the seventh intron of FKBP5 (88). The presence of the T allele of rs1360780 is associated with a higher risk of developing PTSD after child abuse and medical trauma (132); thus, this particular allele is referred to as the “high induction” allele of FKBP5 since it can cause an increase in FKBP5 mRNA levels (132). Conversely, the C allele of rs136780 can cause lower contact of GREs with FKBP5's transcription initiation site and less expression of this gene (132). Controversial studies have shown an increased rate of the T allele in depressive episodes (133) and the C allele in MDD patients (134). Thus, further studies, specifically on MDD patients, are required to determine the exact role of rs136780 in psychiatric disorders.

Rs136780 can also affect the pharmacogenetic aspects of drugs. In the Munich Anti-depressant Response Signature (MARS) project, patients with psychiatric disorders and depressive symptoms who have rs1360780 have shown better responses to treatment with citalopram (135). However, patients who have schizophrenia have a greater probability of not responding to clozapine when they have TT homozygotes of the rs13860780 than the C allele carries (136). However, in patients with rs1360780, no evidence demonstrated a relationship between paroxetine and mirtazapine with treatment outcomes (137). As a result, rs1360780 is an important SNP, and its features for predicting the treatment outcome can be helpful.

### 3.2. Rs9296158

The SNP rs9296158, located in the FKBP5 gene, has been identified as a risk factor for suicide after childhood trauma (31) and the severity of PTSD symptoms (132). Rs9296158 has also been identified as a susceptibility factor for anxiety sensitivity (138) and MDD (112). Patients with cancer who possess rs9296158 after prolonged stress exposure may experience heightened levels of anxiety and depression (139). In addition to the effect of rs9296158 on the expression of FKBP5, this SNP can affect the signal transducer and activator of transcription 5B (STAT5B) mRNA levels. STAT5B is a protein involved in the translocation of GRs to the nucleus of cells. The reduction of STAT5B can cause excessive signaling induced by GRs, exacerbating stress-related effects (140). A recent study has demonstrated that traumatized individuals with the rs9296158 G allele exhibit much more severe dissociation symptoms than those with the rs9296158 A allele (136). Further details outlining the association between rs9296158 and various psychological conditions are presented in Table 2.

### 3.3. Rs4713916

Rs4713916 is associated with MDD (112, 141), psychotic symptoms (142), and higher levels of anxiety after social stressors (143). Rs4713916 can affect MDD patients by decreasing the hippocampal volume and causing a lower response to anti-depressants (112). Moreover, A allele carriers respond better to treatment in patients with mood disorders than those with the G allele (106). In the German population, carriers of the A allele of rs4713916 have a lower incidence of depression (141). Patients could be examined by the Trier Social Stress Test (TSST), measuring the cortisol level after acute stressors to determine the normal and abnormal responses of the HPA axis against acute stress. The result of the TSST can be changed from person to person, because the serum cortisol levels can be genetically influenced by polymorphisms of the FKBP5 gene (144). The result of the TSST in people who have rs4713916, rs380373, and rs1360780 SNPs demonstrated impaired cortisol responses to social stressors. This observation demonstrated that the impaired negative feedback of the HPA axis could result in chronically high levels of GRs and susceptibility to stress-induced psychiatric diseases (143). Finally, more accurate studies are needed to clarify the effect of rs4713916 on the regulation of stress responses.

### 3.4. Rs7748266

The T allele of rs7748266 is associated with an increased risk of attention deficit hyperactivity disorder (ADHD) in children (145), susceptibility to developing depressive symptoms (146), and decreased cortisol levels (111). Rs7748266 is also associated with an increased amygdala reaction in childhood emotional neglect by decreasing the basal cortisol levels (147). The amygdala is the brain's emotional processing center, and its structure could be altered by early life adversity. The amygdala can affect the HPA axis via neurotransmitters that should be noticed in stress-induced psychiatric disorders (148). In addition, rs7748266 is associated with lithium effectiveness and response to stressful situations in bipolar individuals (149). The C allele of rs7748266, by changing the regulation of GR activity, could influence the efficacy of lithium (149).

### 3.5. Rs4713902

Rs4713902 is associated with an increased risk of bipolar disorder, borderline personality disorder, and suicidal behaviors (30, 31, 130). There is also evidence of an association between the C allele of rs4713902 and higher baseline cortisol levels in psychological stress than homozygote T carriers (150). A higher cortisol level may lead to neuropsychiatric disorders by altering neural circuitry (150). GC negative feedback can be reduced when FKBP5 expression or function increases (151). Thus, the improvement in the expression or function of FKBP5 could explain the role of the C allele of rs4713902.

### 3.6. Rs9470079

Martín-Blanco et al. reported that rs9470079 might be involved in the etiology of bipolar disorder despite the polymorphisms associated with bipolar disorder being intronic variants with unknown functions (130). The rs9470079 variant was significantly associated with decreased cortisol suppression, indicating a potential independent relationship between this variant and altered HPA axis negative feedback (152). Previous studies have shown that alleles associated with MDD increase FKBP5 expression, which induces enhanced binding to GR (82, 87, 112), and Ferrer et al. indicated that a similar mechanism might be involved in rs9470079 modifying HPA axis negative feedback as well (152). This decreased cortisol suppression caused by the A allele of rs9470079 can increase the susceptibility of people to stressors (152). Therefore, additional research is needed to identify vulnerable individuals carrying this allele to provide better mental health care.

### 3.7. Rs9394309

In female subjects with breast cancer, certain genetic variations of FKBP5, such as rs9394309, are associated with increased fatigue, depression, and anxiety (153). Rs9394309 is also linked to MDD (112), higher levels of anxiety in female subjects (124), ADHD, and lower cortisol levels in ADHD patients (126). A significant effect on left dorsal amygdala reactivity can be observed in patients with rs9394309 and childhood emotional neglect. This reactivity bias is one reason why people carrying FKBP5 risk alleles (“i.e., rs1360780 T allele, rs9296158 A allele, rs9470080 T allele, rs3800373 G allele, rs7748266 T allele, rs9394309 G allele”) are more likely to suffer from stress-related psychopathologies. In detail, heightened threat-related amygdala reactivity is due to FKBP5 risk genotypes that cause an elevated and prolonged cortisol response to stress (147). However, extreme emotional neglect in childhood can cause higher levels of HPA hyperresponsiveness (154, 155). In addition, anxiety and negative experiences can induce the overexpression of FKBP5 (82, 103, 132). This overexpression of FKBP5 and HPA hyperresponsiveness with adverse experiences (156, 157) can cause HPA negative feedback impairment (158). As a result, both the FKBP5 risk genotypes and a history of emotional neglect are linked to dysregulation of the HPA axis. The interaction of FKBP5 risk genotypes and a history of emotional neglect can increase the amygdala's reactivity and sensitivity to environmental hazards, which increases the risk of stress-related psychopathology (147).

### 3.8. Rs3800373

Rs3800373 is located in the 3′-untranslated region (3′-UTR) of the FKBP5 gene, which may affect the mRNA stability and half-life of FKBP5 (114). SNP rs3800373 CC is more common in patients with MDD and MDD/psychosis. FKBP51 protein levels are higher in these patients than in control groups, but transcription levels do not differ significantly (114). There is a possibility that translation efficiency may be affected by this 3′UTR polymorphism. A study on miRNA binding in 3′UTRs indicates that miRNA binding can influence the stability of mRNA, affecting the half-lives of a specific transcript (159). However, the G allele of rs3800373 can induce the overexpression of the FKBP51 protein, resulting in reduced GR availability and sensitivity to GCs and disrupting the negative feedback loop of the HPA axis. This impaired inhibition of negative feedback can cause susceptibility to psychological diseases (107).

Studies have reported conflicting results about the association of rs3800373 with psychopathology. Binder et al. observed an interaction between childhood trauma and rs3800373, linked to an increased risk of PTSD in African-American populations. However, allelic variation in rs3800373 was not associated with PTSD in an analysis involving predominantly European-Caucasian participants. The results suggest that ethnicity might be an essential factor affecting the functions of the FKBP5 SNPs (160).

According to White et al.'s study, FKBP5 genotypes interacted with childhood emotional neglect to predict increased threat-related dorsal amygdala reactivity. As described in the explanation of the role of rs9394309, individuals with these FKBP5 alleles may be more susceptible to stress-related psychopathology through this reactivity bias in the context of emotional neglect (147). In conclusion, rs3800373 is one of the most important SNPs of the FKBP5 gene, and its association with several diseases has been found before. Moreover, some hypotheses regarding the molecular mechanism of the polymorphism were made before, as were signs about the effect of SNPs on the amygdala. Further research and analysis are required to fully understand the comprehensive role of this SNP and its different alleles.

### 3.9. Rs9470080

The genetic variant rs9470080 has been associated with various mental health conditions such as depressive disorders and symptoms, drug abuse, anxiety disorders, ADHD, rumination disorder, schizophrenia, borderline personality disorder, and PTSD (101, 105, 110, 117, 123, 126, 161). Several studies have indicated that individuals with the rs9470080 TT genotype exhibit reduced FKBP5 mRNA expression, resulting in lower plasma cortisol levels. These patients were more likely to experience depressive and PTSD symptoms after experiencing low-level trauma. Therefore, low cortisol levels may constitute a vulnerability factor that might partially explain the high comorbidity of PTSD and depression. Moreover, the C allele of Rs9470080 appears to play a protective role against developing psychopathologies like PTSD in severe trauma cases. According to Li et al.'s findings, some alleles associated with a certain disorder in one environment may confer protection against that disorder in a different environment and condition (117).

### 3.10. Rs7757037

Rs7757037 is associated with an increased risk for bipolar disorder. The G allele, with higher frequencies in the population, was linked with the risk of bipolar disorder (30). In lupus patients, the FKBP5 gene variant rs7757037 was linked to depression (162), but no similar finding was achieved in an adolescent Chinese population in which the association between depressive symptoms and parenting styles was measured (163).

### 3.11. Rs3798347 and rs10947563

Mahon et al. (150) found an association between rs3798347-T and rs10947563-A alleles and a history of physical abuse and emotional neglect in borderline personality disorder subjects in comparison to patients who had never experienced these traumas and the control group. These two variants are intronic SNPs of the FKBP5 gene. To the best of our knowledge, few studies have been conducted on them, and further studies are needed to determine their exact action.

## 4. Drugs associated with FKBP51

FKBP51 is an essential mediator in the HPA axis of stress responsiveness due to its regulatory effect on GR activity (52, 79). Previous studies have demonstrated how the epigenetic process of DNA methylation is involved in response to psychological therapies, particularly the methylation of FKBP5 at its promoter region, which is associated with PTSD and anti-depressant therapy responses (135, 164). Indeed, the reduction of FKBP5 methylation was strongly correlated with a greater response to therapy (165). Moreover, pharmacogenetic studies on FKBP5 showed promising results in psychopharmacology drug usage (137). FKBP5 can be affected by many substances, and it has been found that many drugs can cross the blood–brain barrier and affect the FKBP51 protein level by methylating the FKBP5 gene (166, 167). The importance of these findings lies in the possible role of FKBP51 as a potential pharmacologic target for stress-induced psychiatric diseases due to its importance in HPA axis regulation (168). In this section, we attempt to describe the relationship between FKBP51 and different drugs and compounds that affect FKBP51 in different aspects. The drugs discussed in this section are the most promising drugs that can inhibit the FKBP51 protein based on our previous *in-silico* study. In a previous study, multiple databases were screened, and those therapeutics that can cross the blood–brain barrier (BBB) and have capable pharmacologic and bioinformatic features for interacting with and inhibiting the FKBP5 gene were reported (43). In addition, the effects of different drugs on FKBP51 are listed in Table 3.

**Table 3:** Research showing the effect of different drugs on FKBP5.

**Type of study**	**Treatment**	**Participants**	**Observations**	**References**
*In vitro*	Citalopram hydrobromide with dexamethasone	Human neuroblastoma cell line	Increased in the FKBP5 mRNA expression	Verjee et al. (169)
Cohort	Citalopram	387 patients with major depressive episodes	Interaction effects between FKBP5 with remission of depressive episodes were found	Horstmann et al. (135)
Randomized controlled trial	Citalopram	1,523 patients with non-psychotic depression	Association between citalopram and FKBP5 expression found	Lekman et al. (133)
Clinical trial	Paroxetine	131 patients suffered from moderate to severe depression	An increase in suicidal ideation within rs1360780 of FKBP5	Perroud et al. (170)
	Venlafaxine			
Cross-sectional	Clozapine	591 cases of schizophrenia	Association between poor response to clozapine and the FKBP5-rs1360780 polymorphism	Mitjans et al. (171)
*In silico*	Enzalutamide	Prostate cancer cell line	The downregulation of FKBP5 can cause enzalutamide resistance	Zheng et al. (172)
*In vivo*	Icariin	50 rats	Icariin can cause the downregulation of the FKBP5 in the hippocampus but not in the prefrontal cortex	Wei et al. (173)
Trial	Prednisolone/liposomal encapsulated prednisolone	Male recipients are injected daily with 5 mg/kg cyclosporine and either 10 mg/kg prednisolone	The FKBP5 upregulation in liposomal-encapsulated prednisolone receptors than prednisolone or no additional treatment group	van Alem et al. (174)
*In vivo*	Dexamethasone	108 mice under stress	Alteration in FKBP5-mediated glucocorticoid sensitivity	Sawamura et al. (175)
Case-control	Dexamethasone	89 MDD cases vs. 126 control group	Correlation between FKBP5 rs9470079-A and a reduced cortisol suppression response to very low dose of dexamethasone	Ferrer et al. (152)
*In vivo*	Dexamethasone	100 rats under unpredictable and stressful situations	Increase FKBP5 level	Xu et al. (176)
*In vitro*	Dexamethasone	Human ovary cells	Significant mRNA increase compared with the control, confirming that the GC receptor was functional and available in human fetal ovaries	Poulain et al. (177)
*In vitro*	Dexamethasone	50 patients' peripheral blood mononuclear cells (PBMC)	FKPB5 expression was induced by dexamethasone	Baptista et al. (178)
*In vitro*	Dexamethasone	Human trabecular meshwork cells	Demethylated the cytosine-phosphate-guanine (CpG) sites within FKBP5 gene promotor region	Matsudaet al. (179)
Case-control	Chlorpromazine	40 cases with first-episode psychosis, 45 cases with schizophrenia (*n* = 85) vs. 56 controls	A decrease in methylation of FKBP5, or indeed a higher expression of FKBP5	Misiak et al. (180)
Cohort	Chlorpromazine	55 cases of schizophrenia and 34 cases of bipolar disorder	An increase in FKBP5 expression	Sinclair et al. (181)

### 4.1. FKBP51 ligands

#### 4.1.1. SAFit1 and SAFit2

SAFits are selective inhibitors of the FKBP51 protein, and their anti-depressant and neurogenic effects have been previously demonstrated (182, 183). Previous studies have shown that SAFits can be utilized for immunosuppression in heart transplant donors by reducing programmed death ligand 1 (PD-L1) (184). Balsevich et al. demonstrated that SAFit2 treatment affects glucose tolerance and reduces body weight, which is specific to FKBP51 inhibition (183).

Besides, in various *in vivo* studies, SAFit2 has also exhibited anti-anxiolytic (185), anti-stress (186), and anti-alcohol consumption (187) effects. Based on a study performed by Pöhlmann et al. (188), it has been revealed that SAFit2 could be a valuable co-medication in the case of depression disorders that could alter the effect of other anti-depressant drugs. In 2014, Gaali et al. (183) used SAFit2 in the combined dexamethasone/corticotropin-releasing factor (Dex/CRF) test to investigate its effect on HPA axis regulation. They reported that SAFit2 improved the elimination of corticosterone levels after applying dexamethasone. Their results are associated with an enhancement of GR sensitivity by FKBP51 inhibition. Researchers demonstrated that FKBP51 inhibitors would improve the regulation of the HPA axis. It has also been noted that the Dex/CRF test could act as a functional biomarker throughout the clinical improvement of selective FKBP51 antagonists (183). However, Connelly et al. proposed that SAFit2 would debilitate stress-induced reinstatement of cocaine seeking in mice through increasing HPA axis negative feedback. Based on this study, SAFit2 would improve not only HPA axis negative feedback managed by GRs but also progesterone receptor sensitivity (189).

While SAFit1 and SAFit2 are two major selective inhibitors of the FKBP51 protein, they are unsuitable therapeutic compounds in clinical and pharmacological aspects. This is because the pharmacological features of these drugs, such as bioavailability and solubility, are far from their pharmacological usage in humans (190). As previously mentioned, finding other drugs for psychological disorder treatment is essential. The promising effects of selective inhibitors of FKBP51 showed the need for developing new pharmacologically suitable drugs for the selective inhibition of FKBP51.

#### 4.1.2. Rapamycin and tacrolimus

Tacrolimus (FK506) and rapamycin (sirolimus) are widely utilized antiproliferative and immunosuppressive drugs that are commonly used to manage cancer and prevent acute graft rejection. They inhibit mTOR activity, which is known for its significant impact on cell growth and proliferation (191, 192). Clinical findings have demonstrated that patients undergoing immunosuppressive drug therapy frequently experience affective disorders such as anxiety or depression. In an investigation by Hadamitzky et al., it was observed that the acute administration of rapamycin upregulated the level of FKBP51 in the brain of mice experiencing psychiatric diseases, while repeated administration did not affect FKBP51 levels (191, 192). Tacrolimus can also bind to immunophilin FK-binding protein 12 (FKBP12) and form FKBP/FK506 complexes that can change the PPIase activity of FKBPs and result in immunosuppressants (193), which indicates the potentiality of tacrolimus to affect FKBP51. To the best of our knowledge, few studies have been conducted on the effect of tacrolimus and sirolimus on psychiatric diseases via FKBP51 modulation. Further research is required to determine the impact of these medications on psychiatric diseases owing to the importance of FKBP51 in stress-induced psychiatric diseases and the binding capacity of these two drugs to FKBP51.

### 4.2. Anti-depressants

#### 4.2.1. Fluoxetine

It has been identified that treatment with selective serotonin reuptake inhibitors (SSRIs) can decrease the activity of CRH neurons and consequently contribute to SSRI's therapeutic activity. It has been propounded that the downregulation of the activity of CRH is the common step of anti-depressant activity. Many investigations have found that anti-depressants of different classes suppress CRH gene expression and HPA activity in depressed and healthy humans (29). In a study on mice exposed to chronic stress, fluoxetine could downregulate the overexpression of FKBP5 mRNA levels in the hippocampus and the prefrontal cortex (173). Besides, in another study that attempted to find an association between fluoxetine efficacy and patients who were carriers of two SNPs of FKBP5, no association was found. However, Maruf et al. (194) demonstrated pharmacogenetic associations between FKBP5 and fluoxetine. However, further research to confirm the absolute gene-antidepressant relationship is needed.

#### 4.2.2. Citalopram

Citalopram is an SSRI whose association between drug response and different FKBP5 SNPs has been studied before (87, 133). In 2010, Zobel et al. (134) selected 110 German patients with unipolar depression after accounting for dropouts and 284 controls. The anti-depressant used was citalopram with or without lorazepam. The anti-depressant response was assessed using a Dex/CRF test. A previous study demonstrated the associations between rs3800373 and rs4713916 and lower citalopram responses (134). Furthermore, only the relation between FKBP5 and Citalopram response in the STAR^*^D sample (135) of the MARS project has been tested to date. A better response to Citalopram was found in a combination of protective SNPs of FKBP5, rs1360780, and glutamate receptor inotropic kainite 4 (GRIK4), rs12800734 (195). Therefore, anti-depressant drugs potentially influence the HPA axis, especially the expression level and genetic alteration of FKBP5. As a result, finding the exact mechanism of action of anti-depressants on FKBP5 should be considered in further research.

#### 4.2.3. Icariin

Icariin, a major component of Herba Epimedii, has been indicated to possess potential anti-depressant effects (159). Icariin could reduce inflammatory cytokines and increase the cortisol level in chronically stressed rats (196). Additionally, in socially defeated mice, the administration of Icariin also renormalized the GR binding affinity and expression levels (197). These results indicated the various effects of Icariin on the HPA axis. Icariin administration in depressed individuals downregulates the FKBP5 gene in the prefrontal cortex and the hippocampus (173), which is similar to the action of anti-depressants such as fluoxetine. This downregulation of FKBP51 is involved in normalizing GR distribution in cells, which may ultimately restore HPA-axis negative regulation (173, 198). While the upregulation of FKBP51 in chronic stress is a normal reaction of the body, FKBP51 blockade could worsen the adverse effects of stress (44). However, the mechanism that explained the role of anti-depressants in the HPA axis in chronic depression was the renormalization of the upregulated FKBP51 level (199).

#### 4.2.4. Paroxetine, venlafaxine, and clomipramine

Paroxetine, venlafaxine, and clomipramine are three anti-depressants, and high levels of any one of the three anti-depressants in the blood were associated with greater suicidal ideation. These anti-depressants could influence FKBP5 expression. The T allele of rs1360780 on FKBP5 was higher in these patients, indicating the involvement of the HPA axis in suicidality (170). As described in a previous study, T alleles of rs136780 are considered “high induction” alleles, resulting in higher FKBP5 mRNA levels (132). As a result, FKBP5 genetic variation could predispose individuals taking anti-depressants such as venlafaxine, paroxetine, and clomipramine to suicidal ideation.

#### 4.2.5. Serotonin and noradrenaline reuptake inhibitors and tricyclic anti-depressants

Duloxetine is an SNRI that serves as an anti-depressant (200). A study conducted in 2013 measured the mRNA and protein levels of FKBP5 in chronic mild stress (CMS). The study found a significant increase in FKBP5 mRNA levels in the ventral and dorsal hippocampus of CMS mice. These changes, which come from CMS, reproduce a depressive-like phenotype. Duloxetine led to controversial results; while it increased FKBP5 mRNA levels in control rats, it controversially decreased the levels of FKBP5 mRNA when administered to CMS rats. These findings suggest that CMS-exposed animals alter FKBP5-GR activity, which is a crucial step in the HPA axis and GR function. It has been proposed that duloxetine treatment can normalize some of these changes (19). Another study on the MDD mouse models revealed that treatment with venlafaxine and sertraline had no impact on the expression of the FKBP5 gene (201). Furthermore, it has been shown that treatment with tricyclic anti-depressants (TCAs) would increase GR binding affinity and GR mRNA expression in rat hypothalamic and hippocampal neurons. These results propose that anti-depressants may enhance GC sensitivity and function, particularly in the brain, and revitalize GR-mediated feedback prevention on the HPA axis (202).

### 4.3. Antipsychotics

#### 4.3.1. Chlorpromazine

Many studies have demonstrated that patients experiencing first-episode psychosis and schizophrenia may exhibit a flattened HPA axis response to stress (203). Although FKBP5 hypermethylation has been detected in patients with schizophrenia (204), Misiak et al. (180) demonstrated that FKBP5 methylation could not be detected after accounting for potential confounding factors. It was suggested that confounders, including medication usage, such as chlorpromazine, could influence FKBP5 methylation and expression in schizophrenia patients. Thus, further investigation of the effects of chlorpromazine on the methylation and expression of FKBP5 should be undertaken in the future.

#### 4.3.2. Clozapine

Clozapine is another atypical antipsychotic drug with a benzodiazepine derivative and is more effective in curing treatment-resistant schizophrenic patients (205). GCs significantly induct the FKBP5-rs1360780 with clozapine consumption in schizophrenia patients. Clozapine is the only FDA-approved drug for treating resistant schizophrenia (TRS), and several variants, such as FKBP5 variants, have been involved in clozapine response. Furthermore, FKBP51 could be a treatment target for clozapine (171).

### 4.4. Corticosteroids and glucocorticoid ligands

#### 4.4.1. Dexamethasone

As HPA axis hyperactivity is the most consistent abnormality in depressive disorders, and GCs maintain and reduce HPA axis activity, dexamethasone as a glucocorticoid agonist potentially positively affects mood disorders (206). A study conducted by Bekhbat et al. reported that an increased gene expression of FKBP5 was associated with depressive symptoms in dexamethasone-administered cases (207). Dexamethasone also upregulates the expression of the FKBP5 gene in osteogenesis, chondrogenesis (208), and choroidal endothelial cells, modulated by the GR (209). Furthermore, hypermethylation of the FKBP5 gene induced by dexamethasone may involve the HPA axis, which has a substantial role in psychotic disorders (210). Dexamethasone can induce FKBP5 mRNA and protein expression (211). Xu et al. (176) also proved that an increase in FKBP5 expression and a decrease in GR expression in the hippocampus and prefrontal cortex had been detected due to dexamethasone administration in adult rats. The potential effects of dexamethasone on FKBP5 expression impact the HPA axis, and its promising antipsychotic effect should be noticed.

#### 4.4.2. Prednisolone

Previous studies have indicated that FKBP51 can be a glucocorticoid sensitivity biomarker due to being the negative regulator of GRs (212, 213). In this regard, a recent study (174) evaluated prednisolone administration's effect on the FKBP5 level. For 24 h, the overexpression of FKBP5 mRNA was found under the effect of liposomal-encapsulated prednisolone (LP). As a result, similar to dexamethasone, the upregulation of FKBP5 can result in GR downregulation. Therefore, the potential effects of dexamethasone could be considered for prednisolone, and further studies are needed.

#### 4.4.3. BI653048

GCs intervene through the GR, a member of the nuclear receptor family of intracellular receptors that are generally placed in the cytoplasm. Recent data indicate that glucocorticoid receptor ligand (GRL) is functionally selective among transrepression and transactivation. These compounds are known as selective glucocorticoid receptor agonists (SEGRAs). Trifluoromethyl carbinol-derived compounds are an extremely well-profiled nonsteroidal class of GRLs. In the study of Harcken et al. (214), a compound named BI653048 was identified. BI653048 is in the SERGA class, which binds with high affinity to the human GR. They revealed that treatment with 200 mg of BI653048 can be associated with a decreased expression of FKBP5 (214).

## 5. Discussion and conclusion

The current study sheds light on the vital role of FKBP5 in stress-induced psychiatric disorders, the HPA axis, and the human body. Despite the limited knowledge of their biological dimensions, it is evident that FKBP5 mutations can significantly contribute to the manifestation and progression of personality disorders and functional seizures by influencing the expression of FKBP51 and the amygdala. The contribution of FKBP5 mutations to many stress-induced psychiatric disorders was found. This significant influence of FKBP5 on stress-induced psychiatric disorders highlights the importance of this gene as a potential drug target. Although the included studies have explored the potential of FKBP51 as a drug target, further investigation is essential to clarify the direct inhibitory effects of the drugs on this gene. If confirmed, psychiatric drugs targeting FKBP51 may not only impact neurotransmitters but also directly modulate the HPA axis. Such significant implications call for pharmaceutical companies to formulate optimized drugs capable of efficiently and simultaneously regulating both neurotransmitters and FKBP51. Hence, the findings from this study establish the significance of FKBP51 as a promising therapeutic target for stress-induced psychiatric disorders.

## Author contributions

MM contributed to study conceptualization, writing, and reviewing. DS and NE contributed to writing and reviewing. MS, SS, MJ, and SM contributed to data acquisition and writing. NA contributed to the study conceptualization and review. All authors contributed to the article and approved the submitted version.
